# Programmable Magnetic Soft Robots via Assembled Magnetization and Joint-Mediated Symmetry Breaking

**DOI:** 10.3390/mi17080927

**Published:** 2026-08-01

**Authors:** Rufei Cui, Boqi Ding, Xiaoyu Zhao, Jiangxing Chen, Yongjun Zhang, Xinyu Wang, Yaxin Wang, Renxian Gao, Kun Zhang, Fengyi Zhang, Zhe Kong

**Affiliations:** 1 College of Materials and Environmental Engineering, Hangzhou Dianzi University, Hangzhou 310018, China; cuirufei@hdu.edu.cn (R.C.);; 2 Department of Physics, Hangzhou Normal University, Hangzhou 311121, China; 3 Division of Microelectronic Materials and Devices, Hangzhou Dianzi University, Hangzhou 310018, China

**Keywords:** magnetic soft robots, programmable magnetization, symmetry breaking, modular assembly, magnetic actuation, directional locomotion

## Abstract

Magnetically actuated soft robots enable untethered operation in confined and complex environments; however, achieving controllable directional locomotion in structurally symmetric systems remains a fundamental challenge due to intrinsic force cancellation under uniform fields. Here, we present a modular strategy that integrates assembled programmable magnetization with energy-biased symmetry-breaking joints to overcome this limitation. By embedding hard-magnetic NdFeB microparticles into an Ecoflex matrix, discrete magnetic units with programmable magnetization are fabricated and assembled into higher-order architectures. We show that asymmetric film constraints prescribe joint polarity and bias strain-energy distribution during actuation, producing distinct deformation modes (folding versus bending) under identical magnetic inputs. This energy asymmetry breaks the balanced response of symmetric structures, enabling net directional motion under spatially uniform magnetic fields. Based on this principle, a segmented crawler achieves a maximum speed of 5.42 mm s^−1^ under a half-wave magnetic field, while a quadruped robot realizes programmable multi-directional locomotion (±X, ±Y) via dual-field coupling, reaching a maximum speed of 3.125 mm s^−1^. These results demonstrate that modular magnetization and joint-mediated energy bias can cooperatively generate controllable directional locomotion through mechanically encoded symmetry breaking. This work provides a scalable design framework for programmable magnetic soft robots under spatially uniform magnetic fields.

## 1. Introduction

Soft robotics has developed rapidly as an interdisciplinary research field by combining compliant materials, structural design, and intelligent actuation strategies to achieve adaptable and safe interactions in complex environments [1,2,3,4,5,6,7]. Compared with conventional rigid robots, soft robots can undergo large and reversible deformation, which allows them to navigate confined spaces, conform to irregular surroundings, and interact gently with biological tissues or fragile objects [8,9,10]. These advantages have stimulated growing interest in their applications in minimally invasive operation [11,12], targeted delivery [13,14], industrial inspection, and search-and-rescue tasks [15]. Among the available actuation methods, magnetic actuation is particularly attractive because it enables wireless control, rapid response, and operation through optically or physically inaccessible media [16,17,18,19,20,21,22]. Recent progress in magnetically actuated soft robots has demonstrated that advances in material design, magnetic programming, and fabrication strategies have greatly expanded the achievable deformation modes and locomotion capabilities of magnetic soft robotic systems [23,24].

The performance of magnetically actuated soft robots strongly depends on how magnetization is programmed within the material. By designing the orientation and spatial distribution of magnetic domains, a wide range of deformation behaviors, including bending, twisting, folding, and shape morphing, can be achieved under external magnetic fields [25,26,27]. To realize such programmability, existing magnetization approaches can generally be classified into global and local strategies [28]. Global methods, such as template-assisted magnetization, are simple and scalable, but their ability to generate complex deformation patterns is limited by the difficulty of controlling magnetization distributions in sophisticated geometries [29,30,31]. In contrast, local or voxel-level programming provides high design freedom and high spatial resolution, enabling more complex three-dimensional transformations [32,33,34,35,36]. However, these approaches typically rely on advanced fabrication techniques, specialized materials, or dedicated equipment, which increase fabrication complexity and limit their broader applicability [37,38]. As an alternative to these methods, assembled magnetization has emerged as a modular strategy that combines programmable magnetic response with relatively simple fabrication [39,40,41], which is conceptually related to recent magnetic-field-assisted assembly strategies for constructing responsive magnetic architectures from discrete building blocks and hierarchical magnetic assemblies [42,43]. Recent studies have demonstrated that modular magnetic soft robots can achieve diverse deformation modes and locomotion behaviors by assembling magnetic units with predefined magnetization profiles, thereby reducing the dependence on complex voxel-level magnetization programming while maintaining programmable functionality [44]. In this approach, prefabricated magnetic units with predefined magnetization orientations are assembled into integrated structures, so that complex overall behavior can be achieved through the arrangement and coupling of discrete modules rather than through material-level reprogramming of an entire body. Compared with these modular magnetic designs, the present work further introduces polarity-dependent mechanical joints to regulate local deformation modes and strain-energy distribution, enabling controllable symmetry-breaking locomotion under spatially uniform magnetic fields through a simple modular assembly strategy. This modular design philosophy provides a practical route for constructing reconfigurable magnetic soft robots with tailored actuation characteristics.

Despite these advances in magnetization programming, achieving controllable locomotion remains a major challenge for magnetic soft robots. In particular, directional locomotion in structurally symmetric systems under spatially uniform magnetic fields is fundamentally hindered by symmetry-induced force cancellation. When the magnetic and mechanical responses of different parts of a robot are symmetrically distributed, the resulting forces and torques tend to balance each other, leading to negligible net displacement even under dynamic actuation. This limitation makes it difficult to realize controllable directional motion without introducing geometric asymmetry or relying on spatially non-uniform magnetic fields. To overcome this difficulty, previous studies have employed various symmetry-breaking strategies, for example by introducing asymmetric body geometries, heterogeneous material distributions, or non-uniform actuation conditions [45,46,47]. Although these methods can generate directional motion, they often depend on permanently asymmetric structural designs or more complex external field control. As a result, their generality and scalability may be limited, especially for modular systems that aim to preserve structural simplicity while retaining controllable locomotion. Therefore, a more accessible strategy is still needed to enable directional motion in otherwise symmetric magnetic soft robots under spatially uniform magnetic actuation.

In this work, we address this challenge by introducing a modular actuation strategy based on joint-mediated energy-biased symmetry breaking. Discrete NdFeB/Ecoflex magnetic units with programmable magnetization orientations are assembled through joints with polarity-dependent mechanical constraints. By prescribing the placement of compliant constraining films at the upper or lower interfaces, the joint response under identical magnetic inputs can be tuned into distinct deformation modes, namely bending and folding. These modes exhibit different deformation amplitudes and unequal strain energy storage, leading to an energetically biased actuation response. This bias provides a physical basis for dynamically breaking the otherwise balanced response of symmetric architectures and enabling net directional motion under spatially uniform magnetic excitation. To demonstrate this concept, we fabricate and characterize NdFeB/Ecoflex composite magnetic units to establish their magnetic, mechanical, and actuation properties. Based on these units, we construct a segmented soft crawler that achieves unidirectional locomotion through joint-level symmetry breaking under a vertical half-wave magnetic field. Furthermore, by coupling vertical oscillating and horizontal bias fields (both spatially uniform), we design a quadruped soft robot capable of programmable multi-directional locomotion along the ±X and ±Y directions. These systems together reveal a consistent relationship among modular magnetization, joint-mediated energy bias, and symmetry-breaking locomotion. This work provides a scalable and experimentally accessible framework for designing magnetic soft robots with programmable directional locomotion, without relying on voxel-level magnetization, multi-material 3D printing, or magnetic field gradients. More importantly, it highlights how mechanically encoded joint asymmetry can cooperate with modular magnetization to generate controllable locomotion, offering a general design principle for multifunctional magnetic soft robotic systems.

## 2. Materials and Methods

### 2.1. Fabrication of Magnetic Units

**Figure 1:**
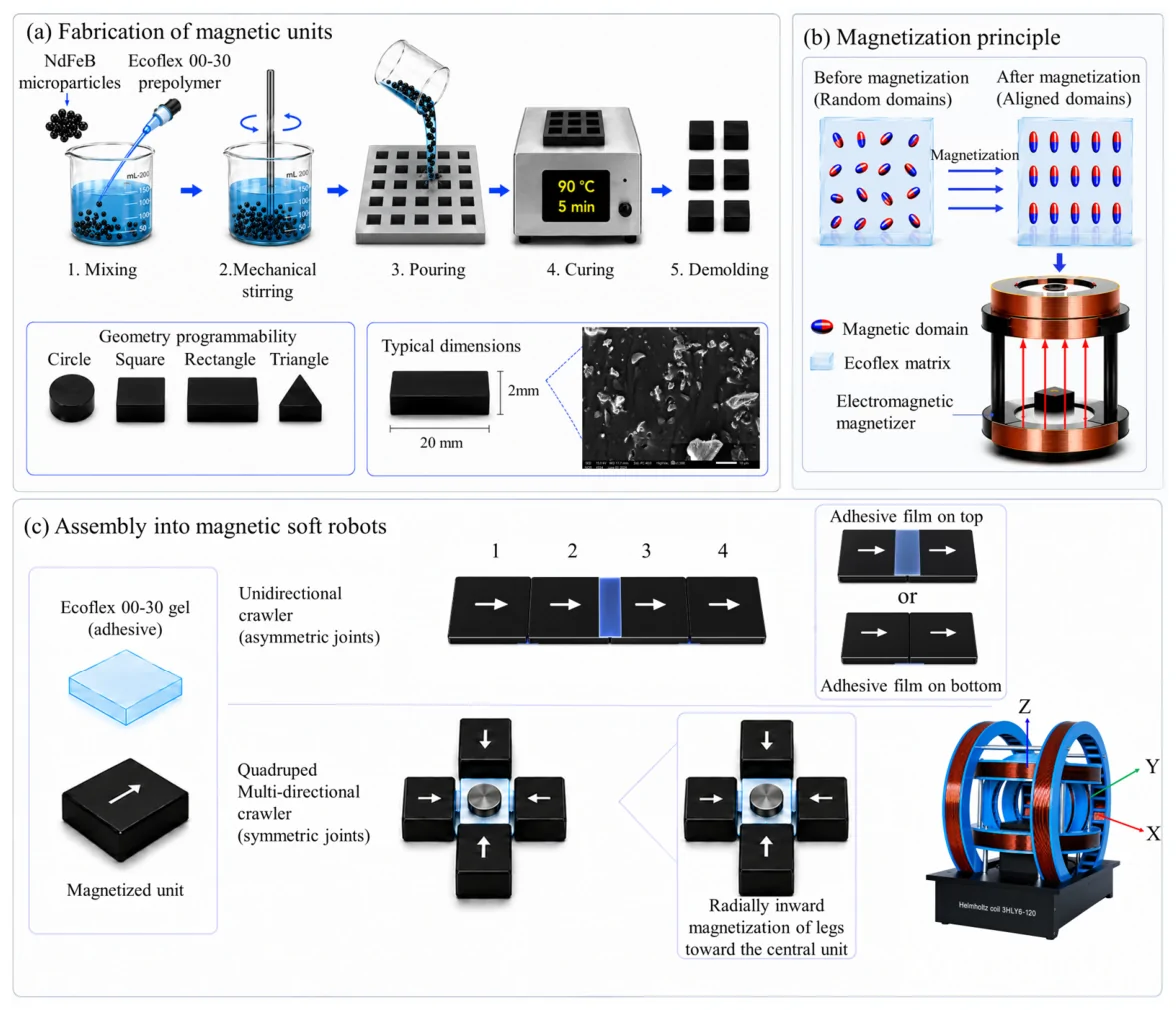
Fabrication, magnetization, and assembly of modular magnetic soft robots. (**a**) Fabrication process of magnetic units. (**b**) Magnetization principle of the magnetic composite. (**c**) Assembly of magnetic units into a unidirectional crawler and a quadrupedal multidirectional crawler, together with the magnetic actuation system.

To enable modular magnetic actuation with programmable deformation responses, composite magnetic units were fabricated by integrating hard magnetic particles with a compliant elastomeric matrix. NdFeB microparticles (average diameter ≈ 5 μm) were selected as the magnetic filler due to their high remanence, which ensures stable and reproducible magnetic actuation. Ecoflex 00-30 (Smooth-On, Inc, Macungie, PA, USA), a platinum-cured silicone elastomer, was used as the matrix material because of its low elastic modulus, high stretchability, and rapid recovery, providing the compliance required for large and reversible deformations under magnetic excitation. This material combination establishes a balance between magnetic responsiveness and structural flexibility, forming the basis for programmable actuation in soft robotic systems. As schematically illustrated in Figure 1a, NdFeB microparticles and Ecoflex 00-30 were mechanically mixed at controlled mass ratios (0.2:1, 0.6:1, and 1.2:1) for approximately 8 min to achieve uniform dispersion and suppress particle aggregation. The resulting mixture was cast into acrylic molds (10 mm × 10 mm × 1 mm), thermally cured at 90 °C for 5 min, and demolded to obtain square composite films. In the as-prepared state, the embedded magnetic particles exhibit randomly oriented magnetic domains, resulting in negligible macroscopic magnetization. To activate magnetic functionality, a programmed magnetization step was performed using an electromagnetic magnetizer (Shenzhen JIUJU Industrial Equipment Co., Ltd., Shenzhen, China). Under an applied external field, the internal magnetic moments are aligned, as illustrated in Figure 1b, thereby generating magnetized units with well-defined magnetization orientations.

The magnetized units were subsequently assembled into higher-order soft robotic structures using Ecoflex 00-30 gel as a compliant adhesive (Figure 1c). During assembly, polarity-dependent joint configurations were introduced by placing compliant constraining layers at different interfaces between adjacent units, thereby imposing asymmetric mechanical constraints within otherwise symmetric structures. Based on this modular design, two representative locomotion systems were constructed: a segmented soft crawler with asymmetric joint connections and a quadruped soft robot consisting of a central body and four peripheral magnetic legs. These joint configurations enable distinct deformation modes under identical magnetic inputs, providing the basis for energy-biased actuation. As a result, the assembled structures can achieve directional and multi-directional locomotion under spatially uniform magnetic fields. This modular assembly strategy not only simplifies fabrication but also offers a scalable and versatile approach for constructing magnetically actuated soft robots with programmable deformation and locomotion capabilities.

### 2.2. Characterization

To elucidate the physical basis of programmable deformation and subsequent symmetry-breaking actuation, the composite magnetic units were systematically characterized in terms of their magnetic, mechanical, and field-induced deformation properties. These characterizations provide the foundation for understanding how magnetic driving forces and elastic resistance collectively determine actuation behavior. Magnetic hysteresis loops were measured using a SQUID VSM (Quantum Design, Inc., San Diego, CA, USA) for samples with different NdFeB:Ecoflex mass ratios (0.2:1 to 1.2:1). The remanence and coercivity obtained from these measurements are directly related to the magnitude of magnetic torque generated under external fields, which governs the driving force for deformation. A TD8620 handheld Tesla meter (TUNKIA Co., Ltd., Wuhan, China) was used to quantify the surface flux density and to verify the field uniformity within the Helmholtz coil center, ensuring consistent and reliable actuation conditions. Mechanical properties were evaluated through uniaxial tensile testing using a ZQ-990 universal testing machine (Outsmart, Shenzhen, China). The elastic modulus was determined from the initial linear region of the stress–strain curve, while the fracture strain was recorded at failure. These parameters quantify the stiffness and deformability of the composites, which define the resistance to magnetic-field-induced deformation. Field-induced deformation behavior was characterized via quasi-static cantilever bending tests, where the tip displacement (D) was measured as a function of the applied magnetic field (B). The deformation sensitivity, defined as ΔD/ΔB (mm mT^−1^), was obtained from the slope of the D–B curve. This metric reflects the efficiency of converting magnetic input into mechanical deformation and provides a direct measure of actuation responsiveness.

### 2.3. COMSOL Simulation

To elucidate the magneto-mechanical deformation behavior of the magnetic units and the strain-energy bias introduced by asymmetric joints, finite element simulations were performed using COMSOL Multiphysics 6.2. Two three-dimensional steady-state magneto-mechanical models were established: (i) a single magnetic unit cantilever model to investigate magnetic-field-induced deformation, and (ii) a two-unit asymmetric joint model to analyze the strain-energy distribution under opposite magnetic-field directions. The simulations were performed by bidirectionally coupling the Magnetic Fields, No Currents (MFNC) and Solid Mechanics interfaces within COMSOL Multiphysics. To accommodate structural deformation during the solution process, the surrounding air domain was defined as a deformable domain with adaptive mesh updating. The model geometry was identical to the experimental specimens. Each magnetic unit had dimensions of 10 mm × 10 mm × 1 mm, while the Ecoflex joint film had a thickness of 0.2 mm. The magnetic composite was modeled as a homogeneous isotropic material with a remanent magnetic flux density of 0.04 T and was assumed to follow an isotropic linear elastic constitutive model. The Young’s modulus and Poisson’s ratio were 50 kPa and 0.45, respectively. For the pure Ecoflex joint film, the Young’s modulus was 4 kPa with a Poisson’s ratio of 0.45. The relative magnetic permeability of both the Ecoflex and the surrounding air was assumed to be 1.

For the magnetic field analysis, magnetic insulation (B⋅n=0) was imposed on the outer boundaries of the air domain to minimize magnetic flux leakage. A spatially uniform static magnetic field with a magnitude ranging from 2 to 10 mT was applied along the ±Z direction as the magnetic actuation field. Continuity conditions were imposed at the interfaces between the magnetic units and the Ecoflex joint film to ensure continuous magnetic flux across the assembled structure. Different mechanical boundary conditions were prescribed for the two simulation models. For the cantilever model, the base surface was fully fixed, whereas the remaining surfaces were left traction-free, allowing deformation to occur solely under the magnetically induced loading. For the asymmetric joint model, a roller boundary condition was imposed on the bottom surface, restricting only the displacement in the z direction while allowing free sliding in the x and y directions to approximate the contact condition between the robot and the substrate. Perfect bonding was assumed at all interfaces to ensure continuous transfer of displacement and stress.

A refined structured hexahedral mesh was employed in the Ecoflex joint region through the film thickness, whereas the surrounding air domain was discretized using free tetrahedral elements. All simulations were performed using a stationary solver with a tolerance factor of 1. The total elastic strain energy of the system was obtained by integrating the elastic strain-energy density over all solid domains,

(1) $$w_{e}=\frac{1}{2}\sigma :\varepsilon$$

(2) $$E_{strain}={∭}_{\Omega_{solid}}w_{e}dV$$

where $w_{e}$ is the elastic strain-energy density, ε is the strain tensor, and σ is the Cauchy stress tensor, and $\Omega_{solid}$ denotes the entire solid domain. During post-processing, the total elastic strain energy of the assembled structure was extracted by performing a volume integration of the strain-energy density (solid.W in COMSOL) over all solid domains. The cantilever model was used to predict the tip deflection under different magnetic field strengths and was quantitatively compared with the experimental bending measurements. The asymmetric joint model was used to evaluate the total elastic strain energy under positive and negative magnetic-field directions, thereby quantifying the strain-energy redistribution and energy-bias mechanism introduced by the asymmetric joint. These simulations provide numerical evidence for the symmetry-breaking mechanism responsible for the directional locomotion of structurally symmetric soft robots under spatially uniform magnetic fields.

### 2.4. Locomotion Performance Evaluation of Soft Robots

Locomotion performance was evaluated in a three-axis Helmholtz coil system capable of generating spatially uniform magnetic fields, ensuring that directional locomotion results from structural and actuation asymmetry rather than field gradients. Field uniformity and flux density at the coil center were verified prior to each experiment using a TD8620 handheld teslameter. For the segmented unidirectional crawler, actuation was driven by a vertical (*Z*-axis) half-wave magnetic field. The waveform of the vertical magnetic field was defined as

(3) $$B_{z}\left(t\right)=B_{max}\left|sin\left(2\pi ft\right)\right|$$

where $B_{max}$ is the peak magnetic flux density (8 mT for the segmented crawler and 4–10 mT for the quadruped robot), f is the actuation frequency (0.5–3 Hz), the DC offset is 0 mT, and the initial phase is 0°. The locomotion performance was quantified by the average crawling velocity (ν), defined as the ratio of a fixed travel distance (10 mm) to the corresponding elapsed time. This metric reflects the effectiveness of converting asymmetric deformation into net directional motion, and thus serves as a direct indicator of symmetry-breaking efficiency. For the quadruped soft robot, programmable locomotion along the ±X and ±Y directions was achieved by superimposing the vertical half-wave magnetic field defined by Equation (3) with a static horizontal bias magnetic field,

(4) $$B_{x}\left(t\right)=B_{bias}\;(or{\;B}_{y}\left(t\right)=B_{bias})$$

where $B_{bias}\leq$ 3 mT. The horizontal bias field remained constant throughout the actuation cycle and therefore exhibited no phase difference with respect to the vertical half-wave magnetic field. The resultant magnetic field was generated by the vector superposition of the vertical oscillating field and the horizontal bias field, enabling simultaneous periodic heaving and directional symmetry breaking. The locomotion performance of the quadruped robot was characterized by the one-step displacement, defined as the center-of-mass translation during a single actuation cycle. To further evaluate dynamic responsiveness, the response time was defined as the interval between the onset of the magnetic field and the maximum warping angle of the actuated limb. Together, these metrics provide a quantitative framework for evaluating how joint-mediated asymmetry and energy-biased deformation are translated into controllable locomotion under spatially uniform magnetic actuation.

## 3. Results and Discussion

### 3.1. Characterization of Magnetic Unit Properties

**Figure 2:**
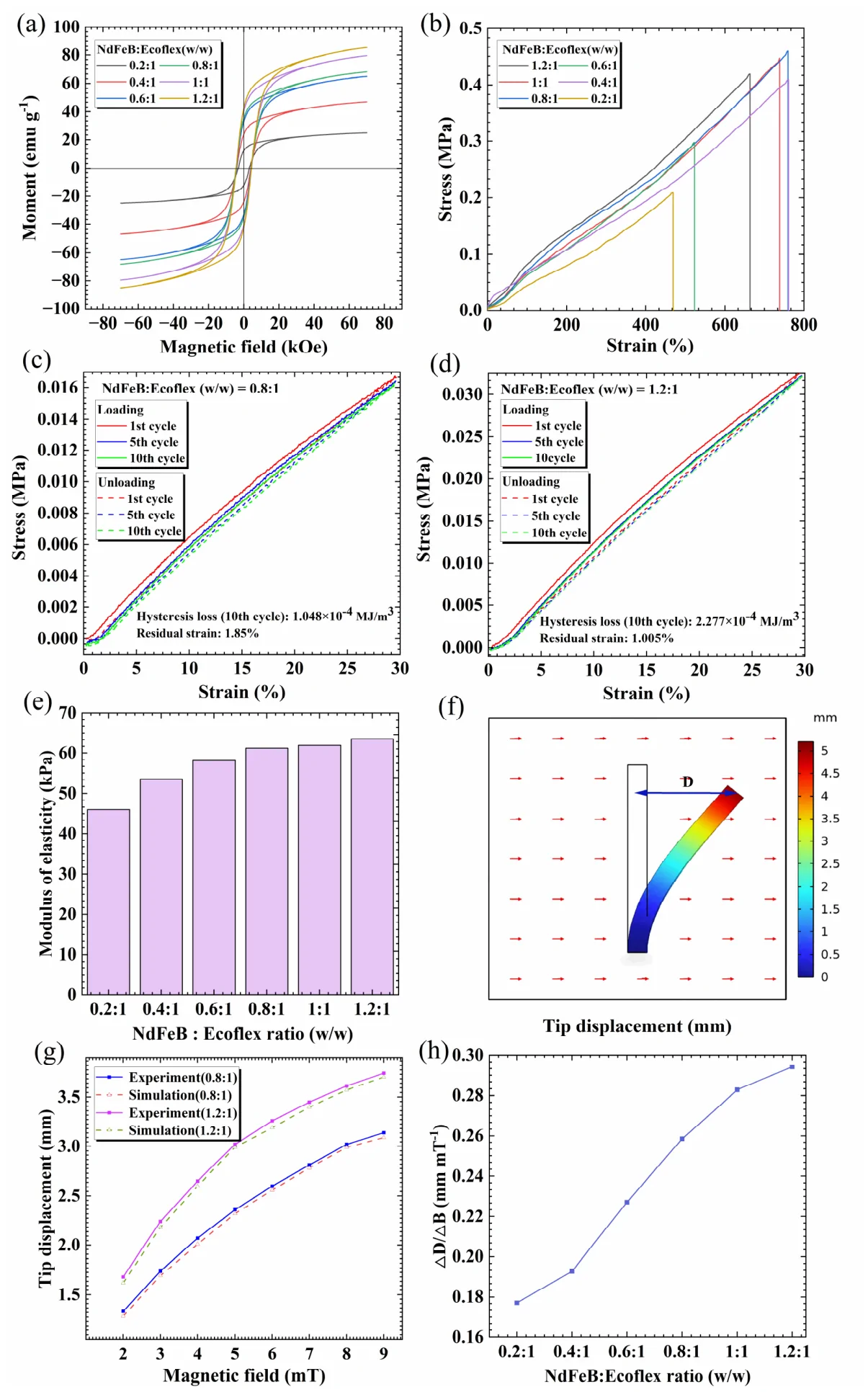
Magnetic/mechanical characterization, numerical validation, and magnetic actuation of NdFeB:Ecoflex units. (**a**) SQUID-VSM hysteresis loops for composites with various mass ratios (*w*/*w*). (**b**) Uniaxial tensile stress–strain curves showing high extensibility across particle loadings. (**c**) Cyclic stress–strain curves over 10 cycles (up to 30% strain) for mass ratios of 0.8:1, indicating low hysteresis loss and minimal residual strain. (**d**) Cyclic stress–strain curves over 10 cycles (up to 30% strain) for mass ratios of 1.2:1. (**e**) Elastic modulus as a function of NdFeB:Ecoflex mass ratio. (**f**) COMSOL simulation profile showing the deformation field (mm) of a magnetic unit under a magnetic field (red arrows). (**g**) Quantitative comparison of tip displacement between experimental measurements and COMSOL predictions vs. field strength (mT) for 0.8:1 and 1.2:1 ratios. (**h**) Deformation sensitivity (ΔD/ΔB) vs. NdFeB:Ecoflex mass ratio.

The actuation of magnetic soft materials is governed by the coupling between their magnetic and mechanical responses, specifically the competition between field-induced magnetic torques and the elastic bending resistance of the polymer matrix. Accordingly, the fraction of magnetic filler relative to the soft elastomer is a decisive design parameter for programmable magnetic systems. SQUID-VSM measurements (Figure 2a) show that the NdFeB/Ecoflex-30 composites exhibit clear ferromagnetic hysteresis with high remanence and coercivity, consistent with hard-magnetic NdFeB imparting permanent magnetization to the matrix. Both saturation magnetization and remanence increase monotonically with the NdFeB:Ecoflex mass ratio, reaching 85.51 emu/g and 43.72 emu/g, respectively, at 1.2:1. Because magnetic torque scales with remanence for a given external field, higher filler loadings afford stronger driving forces for programmable actuation. Uniaxial tensile tests (Figure 2b) indicate that all composites retain excellent extensibility, with fracture strains exceeding 450% and a maximum of 761%, reflecting the high compliance of the Ecoflex-based matrix. To evaluate the elastic recoverability and potential irreversible deformation under working conditions, cyclic loading–unloading tensile tests were conducted at a maximum strain corresponding to the operational regime (30%) (Figure 2c,d). For representative mass ratios of 0.8:1 and 1.2:1, the composites exhibited highly stable stress–strain loops over 10 cycles with negligible hysteresis loss (1.048 × 10^−4^ MJ m^−3^ and 2.277 × 10^−4^ MJ m^−3^ at the 10th cycle, respectively) and minimal residual strains (1.85% and 1.005%). These results confirm that particle incorporation does not induce significant plastic deformation or strain energy dissipation during actuation, ensuring robust elastic recovery. The elastic modulus, obtained from the initial linear region of the stress–strain curve (Figure 2e), increases with particle content and reaches 59 kPa at 1.2:1. This stiffening arises from restricted polymer-chain mobility due to particle inclusion and, at higher loadings, the formation of local particle networks mediated by van der Waals or magnetic interactions. Collectively, these results indicate that although higher particle loading enhances magnetic torque, the associated stiffness increase restricts large-amplitude deformation. To further evaluate field-induced deformation behavior, numerical simulations and bending experiments were conducted (Figure 2f–h). The COMSOL simulation (Figure 2f) illustrates the deformation profile of the magnetic unit under an external magnetic field, highlighting the coupling between magnetic loading and structural response. Quasi-static bending tests were then performed under uniform magnetic fields to quantify the magnetic response of the composites. As shown in Figure 2g, a quantitative comparison demonstrates excellent agreement between the experimentally measured tip displacements and the COMSOL finite element predictions across various magnetic field strengths for both 0.8:1 and 1.2:1 ratios, rigorously validating the accuracy of the numerical model. The maximum deflection increased from 1.33 mm at 2 mT to 3.74 mm at 9 mT for the 1.2:1 ratio, while the deformation magnitude depended strongly on the filler content. Similarly, the deformation sensitivity (ΔD/ΔB), shown in Figure 2h, increased from 0.18 mm mT^−1^ to 0.29 mm mT^−1^ with increasing particle loading, indicating enhanced responsiveness to magnetic stimulation.

Together, these results reveal that the deformation capability of the magnetic units is governed by the balance between magnetic torque and elastic resistance. Although increasing particle loading simultaneously enhances magnetic torque and matrix stiffness, the increase in magnetic responsiveness outweighs the corresponding mechanical resistance, resulting in larger field-induced deformation and higher actuation sensitivity. This balance provides the physical basis for the emergence of distinct deformation modes in assembled structures. As will be demonstrated in the following sections, different joint configurations can exploit this balance to produce bending-dominated and folding-dominated responses with markedly different deformation amplitudes and energy distributions, thereby enabling energy-biased actuation and symmetry-breaking locomotion.

### 3.2. Segmented Unidirectional Crawling Robot

**Figure 3:**
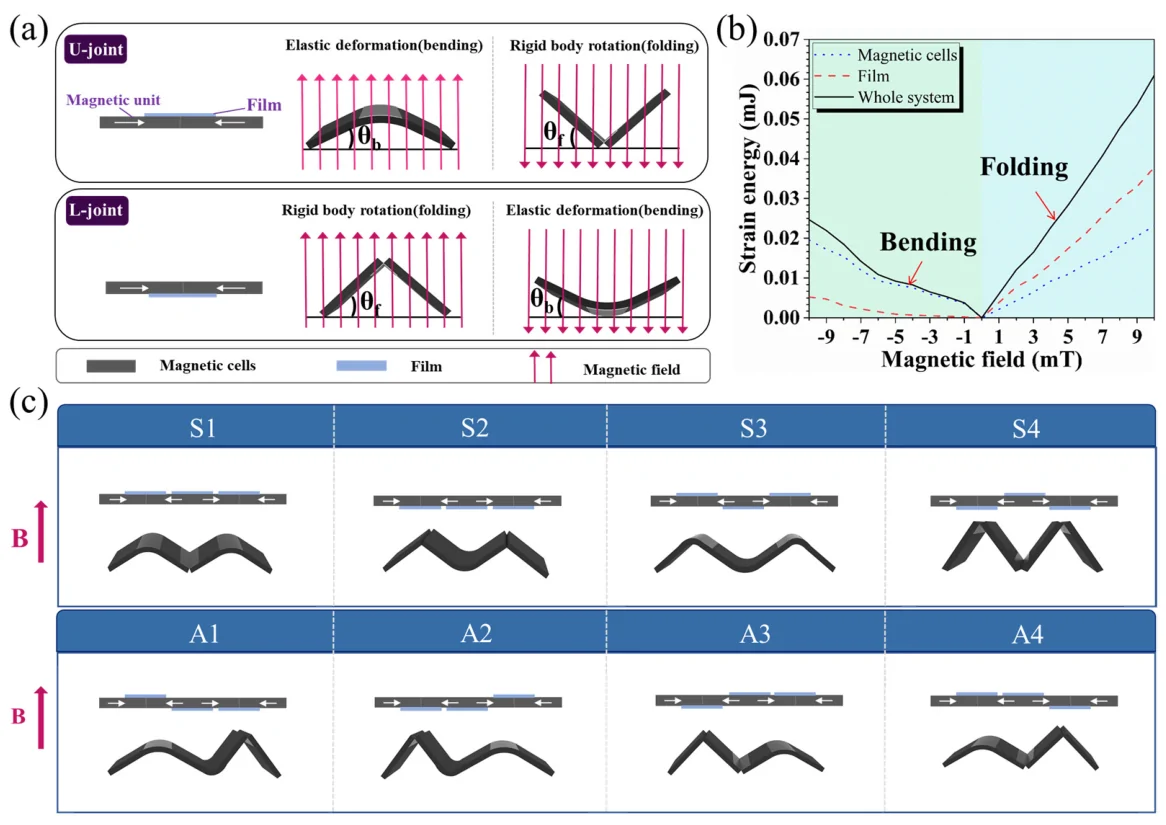
Asymmetric joint design and locomotion principle of segmented unidirectional crawlers. (**a**) Two joint connection modes of magnetic units—upper (“+”, U-joint mode) and lower (“−”, L-joint mode)—and their bending/folding responses under opposite vertical magnetic fields. $\theta_{b}$ and $\theta_{f}$ denote the bending angle and folding angle, respectively. (**b**) COMSOL simulations of the strain-energy distribution in the L-joint mode under positive and negative magnetic fields. The green and cyan shaded regions indicate the negative and positive magnetic field regions, respectively. (**c**) Eight four-unit crawler structures constructed from different asymmetric joint arrangements with symmetric configurations (S1–S4) and asymmetric configurations (A1–A4). The magenta arrows labeled B indicate the applied magnetic field direction, and the white arrows inside the magnetic units indicate their magnetization directions.

To translate unit-level programmability into system-level locomotion, we assembled a strip-shaped segmented crawler by placing magnetized units head-to-head and bridging adjacent units with thin Ecoflex films that act as compliant joints. Depending on the film placement, two joint polarities were defined: upper joints (“+”, film on top) and lower joints (“−”, film on bottom). As shown in Figure 3a, under a vertically upward magnetic field the two jointed pairs exhibited distinct kinematics: in the U-joint mode, the units rotated opposite to the film constraint, producing bending dominated by elastic deformation; in the L-joint mode, the units rotated toward the film, producing folding dominated by quasi-rigid rotation. Reversing the field direction inverted these behaviors. These film-mediated connections, which cause field-direction-dependent responses, are referred to collectively as asymmetric joints. To quantify the underlying energetics, COMSOL simulations (Figure 3b) were performed for the L-joint mode. Under a positive magnetic field, the system folded, with strain energy primarily stored in the connecting film, yielding a total of 0.0609 mJ. Under a reverse field, the system bent, with strain energy primarily stored in the magnetic units, for a total of 0.0246 mJ. These results indicate that, relative to folding, the bending response exhibits higher effective bending stiffness; conversely, under identical actuation, folding stores more energy and attains larger deformation amplitudes than bending. Building on this joint-polarity design, we realized eight four-unit robots by arranging three asymmetric joints (left, middle, and right) in all 2^3^ combinations (Figure 3c). Each four-unit assembly can be conceptually regarded as two dual-unit subsystems coupled in parallel. When the left and right joints were identical (“++” or “−−”), the two subsystems underwent synchronous deformations (S1–S4)—either both bending or both folding—thus preserving center-of-mass symmetry; in this case, the robot merely contracted or extended about its midpoint without producing net translational motion. In contrast, when the left and right joints differed (“+−” or “−+”), the subsystems deformed asymmetrically (A1–A4)—one bending while the other folding—thereby breaking center-of-mass symmetry and inducing a net displacement of the overall system. This demonstrates a symmetry-breaking actuation mechanism that underpins unidirectional crawling in segmented magnetic soft robots.

**Figure 4:**
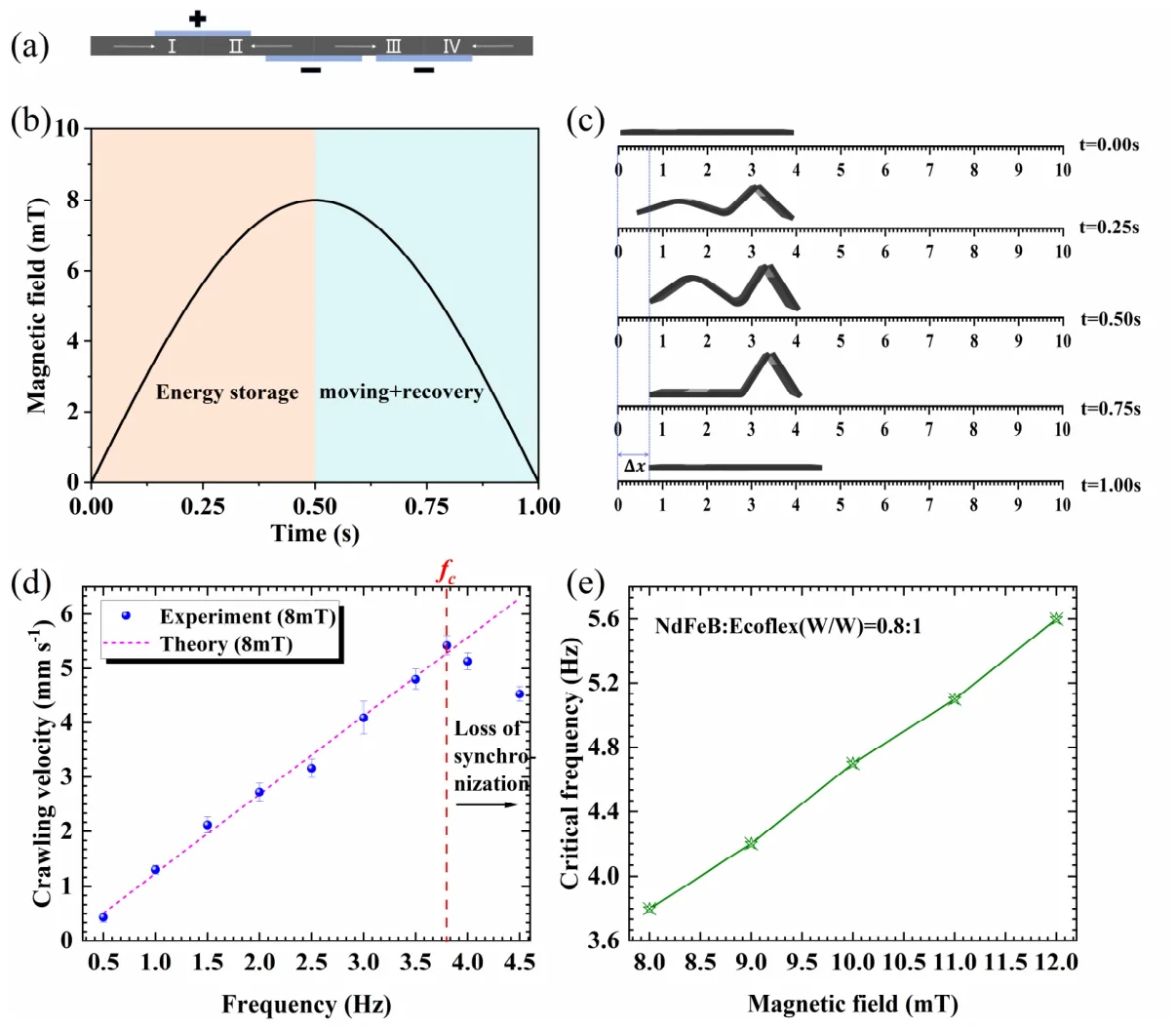
Motion mechanism and performance of the segmented unidirectional crawling robot with a “+−−” joint structure. (**a**) Schematic of the robot configuration. The white arrows indicate the magnetization directions of the magnetic units, the Roman numerals I–IV denote the four magnetic units from left to right. (**b**) Time-domain waveform of the applied driving magnetic field over one cycle. (**c**) Schematic illustration of the crawling mechanism during one complete locomotion cycle (∆x denotes the net forward step). (**d**) Crawling velocity as a function of driving frequency under a fixed field amplitude (8 mT), highlighting the critical frequency ($f_{c}=3.8\;Hz$) beyond which loss of synchronization occurs. (**e**) Critical frequency $f_{c}$ as a function of magnetic field strength (8–12 mT) for the composite ratio of 0.8:1, demonstrating field-enhanced frequency tolerance.

As a representative example, we examined a soft robot with a “+−−” joint structure (Figure 4a). The four sequentially connected magnetic units were denoted as I, II, III, and IV from left to right. Actuation was provided by a vertical (*Z*-axis) half-wave magnetic field, whose waveform over one cycle is shown in Figure 4b. The corresponding crawling mechanism is schematized in Figure 4c. Each locomotion cycle comprised three sequential phases—energy storage, forward translation, and recovery. At the peak field, the left pair of units (I and II) underwent bending, whereas the right pair (III and IV) underwent folding. Because folding produces a larger deformation amplitude than bending, the motion of units III and IV effectively pulled units I and II forward through the compliant inter-unit joints. As the field decreased from its maximum, the units relaxed; when the field reached zero, the bent units recovered first and established broad contact with the substrate, generating high static friction. This anchoring prevented backward slip during recovery and yielded a net forward step of the entire crawler, as demonstrated in Video S1 (Supporting Information). Figure 4d plots crawling velocity as a function of driving frequency at a fixed field amplitude (8 mT). In the low-frequency regime (0.5–3.8 Hz), the speed increases linearly with frequency and shows good agreement with theoretical predictions, reaching a maximum of 5.42 mm s^−1^ at 3.8 Hz. This linear enhancement arises because higher-frequency actuation generates more deformation cycles per unit time without sacrificing single-cycle displacement. However, as the actuation frequency exceeds a critical threshold ($f_{c}=3.8\;Hz$), the crawling velocity rapidly declines, marking a transition into a desynchronized regime (“Loss of synchronization”). This behavior occurs because the mechanical recovery of the viscoelastic elastomer matrix can no longer keep pace with the rapidly alternating magnetic field, resulting in incomplete cycle deformation and reduced net forward displacement. To further explore the field-parameter dependence, the critical frequency $f_{c}$ was evaluated across various magnetic field strengths (8–12 mT) (Figure 4e). The results demonstrate that $f_{c}$ increases monotonically with field strength, rising from $f_{c}=3.8\;Hz$ at 8 mT to 5.6 Hz at 12 mT. A higher magnetic field strength provides a larger field-induced magnetic torque, which enables the soft structure to overcome dynamic phase lag and maintain synchronous actuation at elevated frequencies.

### 3.3. Quadruped Multi-Directional Crawling Soft Robot

**Figure 5:**
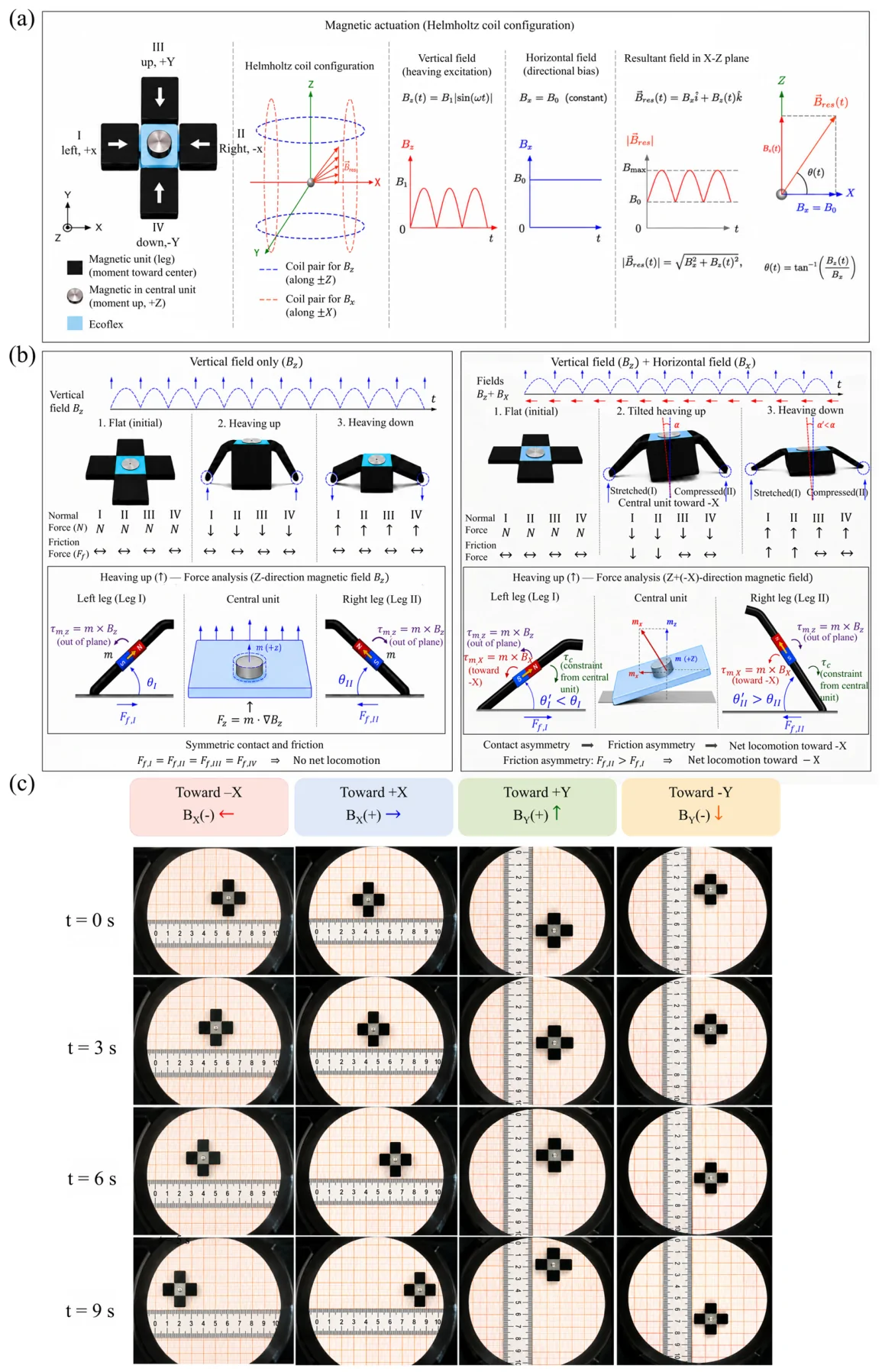
Design principle and programmable multidirectional locomotion of the quadrupedal magnetic soft robot under coupled uniform magnetic fields. (**a**) Magnetic actuation scheme and generation of the resultant magnetic field through the superposition of a vertical oscillating field and a horizontal bias field. (**b**) Comparison of robot deformation behaviors and corresponding force analyses under a vertical magnetic field alone and under combined vertical and horizontal magnetic fields, revealing the symmetry-breaking mechanism responsible for directional locomotion. Red and blue arrows in the top row indicate the horizontal ($B_{X}$) and vertical ($B_{Z}$) magnetic fields, respectively, and blue circles indicate the leg–substrate contact points. (**c**) Experimental demonstrations of programmable crawling toward the −X, +X, +Y, and −Y directions by adjusting the horizontal bias field.

Building on the symmetry-breaking actuation principle demonstrated in the segmented unidirectional crawler, a quadruped soft robot capable of programmable multidirectional locomotion was developed. As shown in Figure 5a, the robot consists of four magnetic legs (Legs I–IV) arranged along the −X, +X, +Y, and −Y directions and a central control unit containing an embedded cylindrical permanent magnet. The magnetic moments of the four legs are programmed radially inward toward the central unit, whereas the magnetic moment of the embedded cylindrical magnet is oriented along the +Z direction. This magnetization configuration enables the robot to generate periodic heaving deformation under a vertical oscillating magnetic field and directional locomotion under coupled magnetic fields. Figure 5a further illustrates the magnetic actuation strategy. A vertical half-wave magnetic field $B_{Z}(t)$ provides periodic heaving excitation, while a constant horizontal magnetic field ($B_{X}$ or $B_{Y}$) serves as a directional bias field. The superposition of these two orthogonal magnetic fields produces a resultant magnetic field with a time-varying magnitude and orientation, enabling simultaneous vertical actuation and directional steering.

To reveal the locomotion mechanism, the robot behavior was first examined under a vertical magnetic field alone. As shown in the left panel of Figure 5b, all four legs bend upward synchronously when the magnetic field increases, producing a symmetric heaving motion. Meanwhile, the embedded central magnet experiences an upward magnetic force along the +Z direction, further contributing to the overall lifting of the robot. As the magnetic field decreases, the elastic restoring force drives the structure back to its initial flat state. Owing to the structural and magnetic symmetry, all four legs exhibit nearly identical deformation amplitudes and contact conditions throughout the actuation cycle. Consequently, the friction forces acting on the four legs remain balanced,

(5) $$F_{f_{,}I}=F_{f_{,}II}=F_{f_{,}III}=F_{f_{,}IV}$$

resulting in zero net displacement despite the large cyclic deformation. To generate directional locomotion, the symmetric deformation mode must be intentionally broken. This is achieved by introducing a horizontal bias magnetic field. Importantly, the robot remains geometrically symmetric throughout the process, while the symmetry is dynamically broken by the coupling between the horizontal bias field and the mechanically constrained central unit. Taking locomotion toward the −X direction as a representative example, the right panel of Figure 5b illustrates the coupled deformation mechanism under the simultaneous application of $B_{Z}$ and $B_{X}$(−X). Under the horizontal bias field, the embedded cylindrical magnet within the central unit tends to rotate from its original +Z orientation toward the −X direction. This rotation causes the central unit to tilt leftward, thereby introducing a mechanical coupling effect that redistributes the deformation of the surrounding legs. For Leg I, whose magnetic moment points toward the central unit (+X direction), the horizontal bias field tends to rotate the leg toward the −X direction. However, the leftward tilt of the central unit constrains this deformation, leading to a reduced warping angle and weaker substrate contact. In contrast, the deformation of Leg II is enhanced by the tilted central unit, resulting in a larger warping angle than that observed under the vertical field alone. Consequently, an asymmetric warping-angle distribution is established,

(6) $$\theta_{I}^{\prime }<\theta_{I},\;\theta_{II}^{\prime }>\theta_{II}$$

where $\theta_{I}$ and $\theta_{II}$ denote the warping angles under the vertical magnetic field alone, whereas $\theta_{I}^{\prime }$ and $\theta_{II}^{\prime }$ represent the corresponding warping angles under the coupled magnetic fields. The resulting deformation asymmetry produces unequal contact conditions with the substrate. Leg II maintains stronger contact and therefore experiences a larger friction force, whereas Leg I exhibits weaker contact and lower friction, yielding

(7) $$F_{f,II}>F_{f,I}$$

During the subsequent heaving-down phase, this friction imbalance is converted into net propulsion toward the −X direction. In contrast, Legs III and IV are oriented perpendicular to the bias-field direction and therefore experience only minor deformation differences. Their friction forces remain approximately balanced,

(8) $$F_{f,III}\approx F_{f,IV}$$

effectively suppressing lateral drift and improving directional locomotion efficiency. Therefore, the horizontal bias field acts primarily as a symmetry-breaking trigger rather than a direct propulsion source. Directional locomotion arises from the friction asymmetry generated by coupling-induced warping-angle differences between the legs.

As described above, the −X direction is selected as a representative example to illustrate the locomotion mechanism. Owing to the rotational symmetry of the quadruped architecture, simply reorienting the horizontal bias magnetic field toward the +X, +Y, or −Y directions enables programmable locomotion toward the corresponding directions without modifying either the robot structure or the magnetization configuration. As experimentally demonstrated in Videos S2–S5, the quadruped soft robot achieves reversible and programmable multi-directional crawling under spatially uniform magnetic fields, highlighting the effectiveness of the proposed magnetic–mechanical symmetry-breaking strategy for untethered soft robotic locomotion.

**Figure 6:**
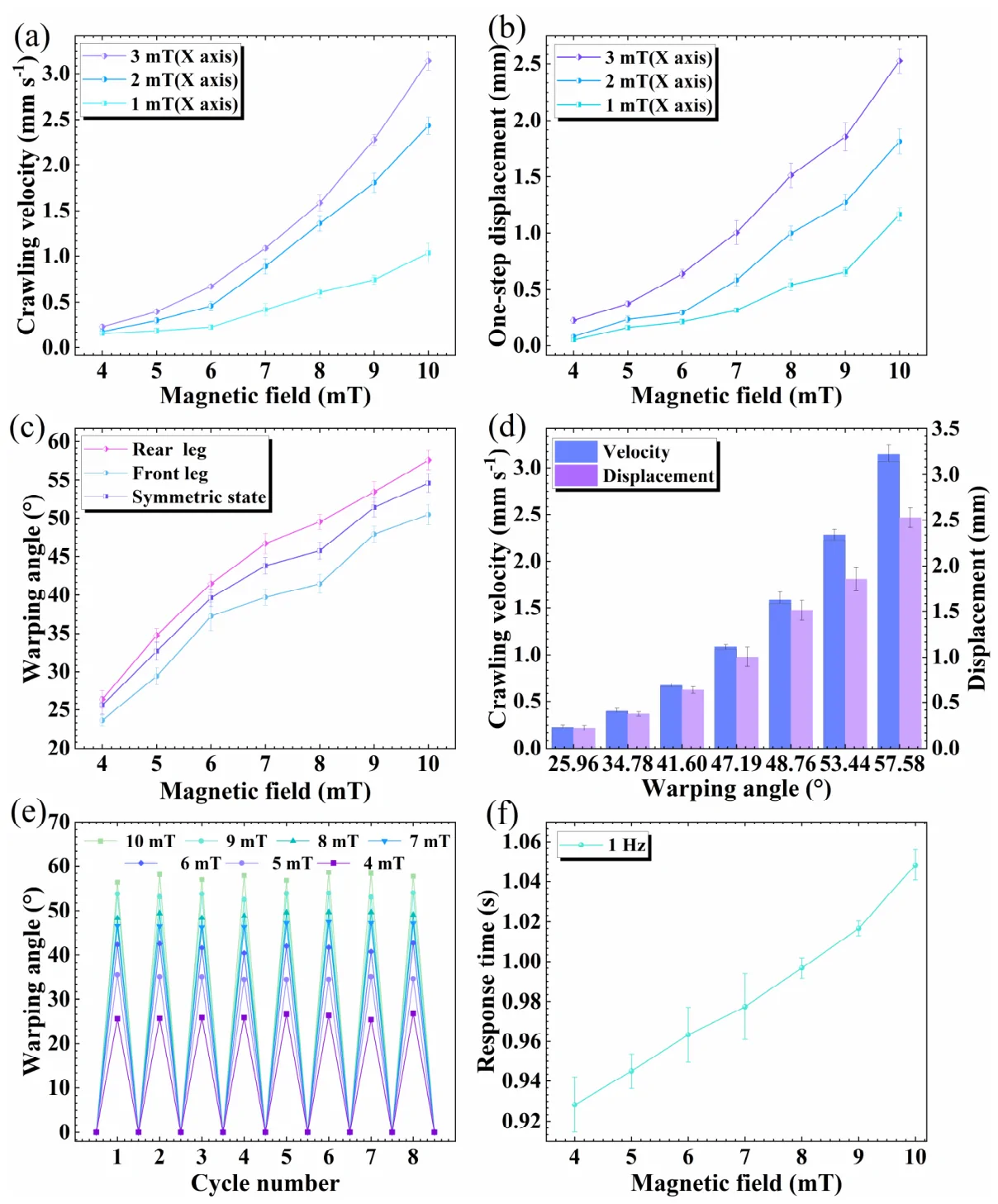
Characterization of the locomotion performance of the quadruped multi-directional soft robot. (**a**) Crawling velocity as a function of vertical magnetic field strength under different horizontal fields (+X axis). (**b**) One-step displacement as a function of vertical magnetic field strength under different horizontal fields (+X axis). (**c**) Variation in maximum warping angles of the front and rear legs under a vertical field combined with a 3 mT horizontal field, compared with the symmetric state (no horizontal field). (**d**) Correlation between crawling velocity/one-step displacement and the maximum warping angle of the rear leg. (**e**) Repeatability of the rear leg’s maximum warping angle over eight actuation cycles under different field strengths. (**f**) Response time of the robot as a function of applied magnetic field strength. All data are presented as mean ± SD (*n* = 3 independent measurements).

The movement of the robot was governed by the coupled effect of the vertical oscillating field and the horizontal bias field. Figure 6a,b show the crawling velocity and one-step displacement as functions of the vertical magnetic field strength under different horizontal bias fields applied along the +X direction. As the vertical magnetic field increased from 4 to 10 mT, both the crawling velocity and one-step displacement increased monotonically under all tested horizontal bias fields, indicating that stronger vertical excitation produced larger heaving deformation and more effective locomotion. Among all tested conditions, a horizontal bias field of 3 mT yielded the best locomotion performance, where the maximum crawling velocity reached 3.125 mm s^−1^ and the maximum one-step displacement reached 2.5 mm. To further clarify the origin of this performance enhancement, the warping angles of the front and rear legs were measured under a 3 mT horizontal bias field and compared with the symmetric state without horizontal bias, as shown in Figure 6c. The results clearly demonstrate that the horizontal bias field breaks the symmetric deformation mode. The rear leg exhibits a larger maximum warping angle than that in the symmetric state, whereas the front leg shows a smaller warping angle. This observation is consistent with the symmetry-breaking mechanism proposed in Figure 5, where the tilted central unit redistributes the deformation of the two legs through mechanical coupling. Consequently, the rear leg contributes more significantly to propulsion, whereas the front leg maintains weaker contact with the substrate. This warping-angle asymmetry leads to an imbalance in normal forces and friction forces during the heaving cycle, thereby generating net displacement.

To verify the relationship between leg deformation and locomotion performance, the crawling velocity and one-step displacement were measured as functions of the maximum warping angle of the rear leg, as shown in Figure 6d. Both metrics increased with increasing rear-leg warping angle, confirming that locomotion performance is positively correlated with rear-leg deformation. Figure 6e further demonstrates the repeatability of the deformation over eight successive actuation cycles. The maximum warping angles of rear legs remained highly stable during repeated actuation, demonstrating reliable cyclic deformation under magnetic control. In addition, the response time of the robot was measured under different magnetic field strengths, as shown in Figure 6f. Although the response time exhibited a slight increase with increasing magnetic field strength, it remained as short as 1.05 s even at the highest applied field, indicating rapid and robust responsiveness. Overall, these results demonstrate that the proposed magnetic–mechanical symmetry-breaking strategy not only enables programmable directional locomotion but also provides stable, repeatable, and highly responsive actuation performance.

## 4. Conclusions

In summary, this work addresses the locomotion limitations of structurally symmetric soft robots through a programmable assembly strategy based on symmetry-breaking actuation. Our characterization reveals that although increasing particle loading simultaneously enhances magnetic torque and matrix stiffness, the improvement in magnetic responsiveness dominates the overall deformation behavior. By combining assembled programmable magnetization with joint-mediated energy-biased actuation, both unidirectional and programmable multidirectional locomotion are achieved under spatially uniform magnetic fields. These results demonstrate that complex locomotion can be realized through the strategic arrangement of modular magnetic units and mechanically encoded constraints, bypassing the need for voxel-level magnetization programming or multi-material fabrication. These findings establish a scalable framework for programmable magnetic soft robots and provide a general design strategy for achieving controllable locomotion through modular magnetization and mechanically encoded symmetry breaking. Looking forward, this modular, symmetry-breaking mechanism establishes a scalable framework that extends the operability of soft robots into highly restricted, unstructured, or dynamic environments, promising for applications such as targeted cargo delivery in tortuous biological lumina or exploration in confined microfluidic channels. To successfully bridge the gap between this laboratory-scale proof of concept and practical engineering deployment, further advancements are envisioned across three intertwined frontiers. First, regarding material design, integrating stimuli-responsive smart matrices (e.g., shape-memory polymers or transient elastomers) will allow the robots to adaptively modulate their local compliance or reversibly reconfigure their modular components in situ. Furthermore, a systematic characterization of long-term mechanical fatigue across different polymer formulations will be crucial for evaluating cyclic durability and preventing joint failure over multi-hundred-cycle operations. Second, in terms of structural design, expanding from planar linkages to multi-layered or closed-loop mechanical joints will unlock more sophisticated, multi-modal locomotion regimes. Finally, advancing magnetic-field control from open-loop actuation to intelligent, closed-loop algorithmic control with real-time imaging feedback will ensure robust navigation through uncertain or highly viscous media. Collectively, these future trajectories will firmly solidify this general design principle, transforming modular soft robots into resilient, adaptive systems capable of real-world deployment.

## Data Availability

The original contributions presented in this study are included in the article/supplementary material. Further inquiries can be directed to the corresponding authors.

## Supplementary Materials

The following supporting information can be downloaded at https://www.mdpi.com/article/10.3390/mi17080927/s1. Supplementary Text: S1. Substrate Friction Characterization and Locomotion Performance and S2. Trajectory Repeatability and Path-Following Precision. Video S1: Segmented crawler locomotion. Video S2: Quadruped locomotion along the +X direction. Video S3: Quadruped locomotion along the −X direction. Video S4: Quadruped locomotion along the +Y direction. Video S5: Quadruped locomotion along the −Y direction.
