# Supplementary material for: Programmable Magnetic Soft Robots via Assembled Magnetization and Joint-Mediated Symmetry Breaking
Source: Micromachines (Basel). 2026 Aug 1;17(8):927. doi: 10.3390/mi17080927 (PMC13515808; doi:10.3390/mi17080927)
Supplement: Supplementary file 1 [file micromachines-17-00927-s001.zip › Supplementary Text.pdf]

Supplementary Information

Programmable Magnetic Soft Robots via Assembled Magnetization and Joint-Mediated Symmetry Breaking

Rufei Cui [1], Boqi Ding [1], Xiaoyu Zhao [1,*], Jiangxing Chen [2], Yongjun Zhang [1], Xinyu Wang [1], Yaxin Wang [1], Renxian Gao [1], Kun Zhang [1], Fengyi Zhang [1], Zhe Kong [3,*]

[1] College of Materials and Environmental Engineering, Hangzhou Dianzi University, Hangzhou, Zhejiang 310018, China
[2] Department of Physics, Hangzhou Normal University, Hangzhou, Zhejiang 311121, China
[3] Division of Microelectronic Materials and Devices, Hangzhou Dianzi University, Hangzhou, Zhejiang 310018, China
* Correspondence: zhaoxy@hdu.edu.cn (X.Z.); zhekong@hdu.edu.cn (Z.K.).

S1. Substrate Friction Characterization and Locomotion Performance

To experimentally investigate the influence of substrate friction on locomotion performance and evaluate the robustness of the proposed locomotion strategy, experiments were conducted on two representative substrates with distinctly different frictional characteristics, namely an acrylic plate and paper. The static friction coefficient of each substrate was determined using the inclined-plane method according to

$$\mu_s = \tan\theta_c$$

where θ_c is the critical inclination angle at which the robot begins to slide. As shown in Figure S1a, the acrylic plate exhibited a critical inclination angle of 20.17°, corresponding to a static friction coefficient of 0.367, whereas the paper substrate exhibited a larger critical angle of 30.90° and a higher static friction coefficient of 0.598. Compared with the acrylic plate, the static friction coefficient of paper increased by approximately 63%, providing substantially stronger frictional support during locomotion.

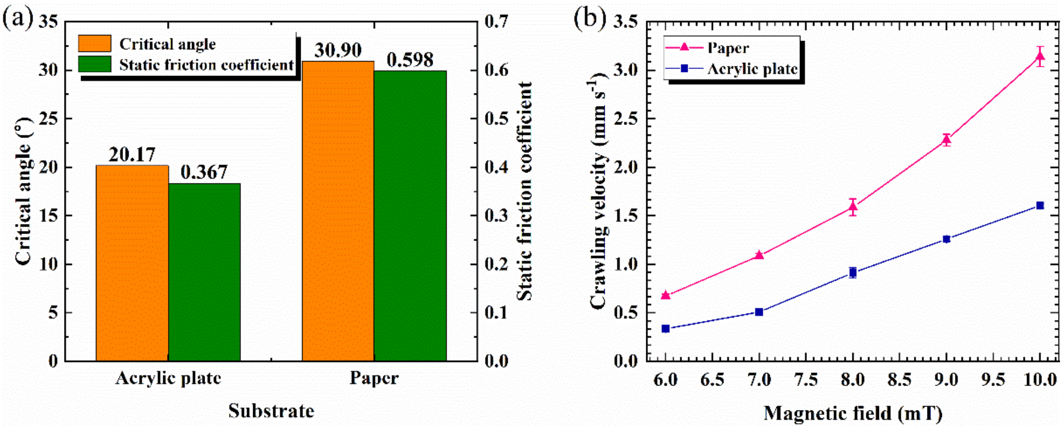

Figure S1. Substrate friction characterization and locomotion performance on different substrates. (a) Critical inclination angle and corresponding static friction coefficient measured on the acrylic plate and paper using the inclined-plane method. (b) Crawling velocity as a function of magnetic-field strength on the two substrates. Each data point represents the mean ± SD of three independent measurements (n = 3).

The locomotion performance of the robot was subsequently evaluated under magnetic fields ranging from 6.0 to 10.0 mT. Each experimental condition was repeated three times, and the results are

presented as mean ± standard deviation. As shown in Figure S1b, the robot achieved stable directional locomotion on both substrates throughout the investigated magnetic-field range. For both substrates, the crawling velocity increased monotonically with increasing magnetic-field strength, indicating that stronger magnetic actuation generated larger body deformation and consequently improved propulsion performance. Quantitatively, the average crawling velocity on the acrylic plate increased from 0.336 ± 0.005 mm s^{-1} at 6.0 mT to 1.606 ± 0.023 mm s^{-1} at 10.0 mT, representing an approximately 4.8-fold increase. On the paper substrate, the average velocity increased from 0.673 ± 0.021 mm s^{-1} to 3.140 ± 0.103 mm s^{-1}, corresponding to an approximately 4.7-fold increase. At an intermediate magnetic field of 8.0 mT, the robot achieved average crawling velocities of 0.918 ± 0.022 mm s^{-1} on the acrylic plate and 1.587 ± 0.086 mm s^{-1} on paper. Across the entire magnetic-field range, the crawling velocity on paper consistently exceeded that on the acrylic plate. At the maximum magnetic field of 10.0 mT, the velocity on paper reached 3.140 mm s^{-1}, approximately 1.95 times that obtained on the acrylic plate.

The higher locomotion efficiency observed on the paper substrate indicates that an increased static friction coefficient enhances the anchoring capability of the supporting legs during the stick phase of locomotion, thereby suppressing backward slipping and improving the conversion of cyclic body deformation into effective forward displacement. Consequently, the paper substrate consistently exhibited superior locomotion performance over the entire range of magnetic-field strengths. More importantly, despite the substantial difference in substrate friction, the robot maintained stable, repeatable, and controllable directional locomotion on both substrates throughout the investigated magnetic-field range. The monotonic increase in crawling velocity with magnetic-field strength, together with the relatively small standard deviations obtained from repeated measurements, demonstrates the excellent repeatability of the experimental results. These findings experimentally verify the important role of substrate friction in the proposed stick-slip locomotion mechanism while confirming that the joint-mediated symmetry-breaking strategy enables robust and reliable locomotion across substrates with different frictional properties.

S2. Trajectory Repeatability and Path-Following Precision

To further evaluate the spatial motion repeatability and controllability of the proposed robot under cyclic magnetic actuation, trajectory tracking experiments were conducted under identical magnetic actuation conditions. Three parallel straight-line trajectories ($Y = 2.6\text{ mm}, 2.8\text{ mm}, 3.0\text{ mm}$) were prescribed as target paths. As depicted in Figure S2, the robot trajectories are represented in a Cartesian coordinate system, where the horizontal axis denotes forward position (X position) and the vertical axis denotes lateral position (Y position). Over a total travel distance of $X = 40$ mm, the experimentally measured trajectories (blue solid lines with square markers) closely match the target desired trajectories (dashed lines) with negligible lateral deviations. The absence of side-drifting confirms that the robot possesses exceptional trajectory repeatability and robust path-following capability, further verifying the reliability of the proposed stick-slip locomotion mechanics.

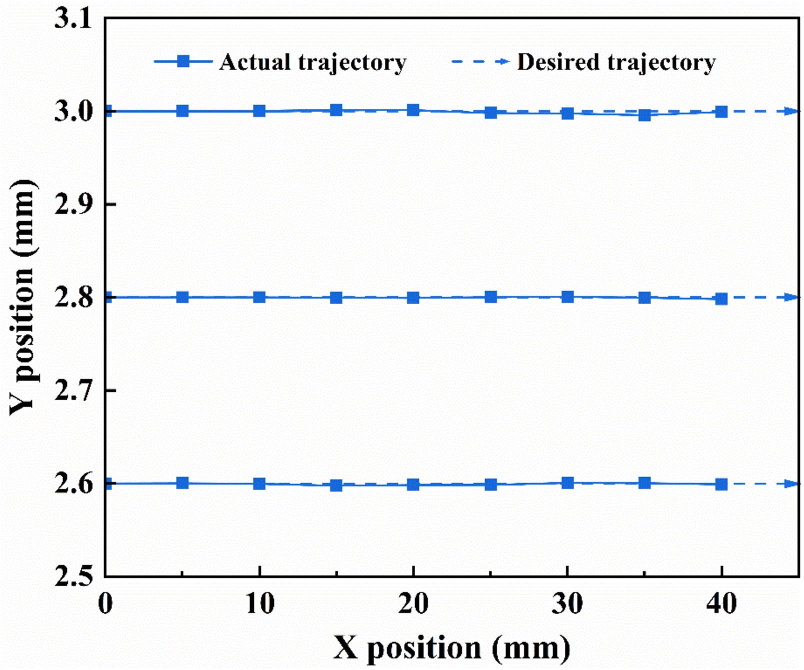

Figure S2. Experimental validation of trajectory repeatability. Comparison of actual measured positions against three parallel desired paths (Y = 2.6 mm, 2.8 mm, 3.0 mm) over a forward displacement of 40 mm in a Cartesian coordinate system.
